# Parliament and the Profession

**Published:** 1916-11-04

**Authors:** 


### PARLIAMENT AND THE PROFESSION.

### Discharged Soldiers—Mental Cases.

Mr. Forster stated that up to the end of August 396 men had been discharged, excluding cases in no way due to war service, owing to the effects of uncertifiable loss of balance, or sent direct to lunacy institutions; he informed Mr. Byrne that committees of these institutions regulate their charges with a view to the needs of the patients' families, and in cases of hardship forgo charges altogether.

### Treatment and Instruction of Disabled Soldiers.

Several questions were put to Mr. Hayes Fisher in respect of the delay in forwarding a scheme for the training of partially disabled soldiers to follow suitable employment, and the consequent interruption in the work of restoring these men to complete health. The answer given was that this was one part of general questions at present under consideration between the Army Council and the Statutory Committee.

### National Insurance Act.

Mr. Roberts informed Sir Philip Magnus that panel practitioners can, in appropriate circumstances, sue insurance committees for alleged breach of agreement, but that the Insurance Commissioners, in respect of certain matters, had, at the request of the medical profession, accepted the responsibility of adjudicating between committees and doctors. Mr. Roberts said that he had no evidence that this procedure had not given general satisfaction.

### Venereal Diseases.

Mr. Hayes Fisher informed Colonel Norton Griffiths that his attention had been called to various resolutions passed by public bodies for Government action in regard to the increase of venereal disease. He stated that definite action had already been taken on the lines of the recommendations of the Royal Commission, and that he was satisfied that best progress is likely to be made by following the advice of the Commission that no system of notification shall be put in force at the present time.

### T.N.T. Poisoning of Munition Workers.

In respect of two recent deaths of female munition workers by T.N.T. poisoning, Mr. Brace, for the Home Office, informed Lord Henry Cavendish-Bentinck of the circumstances of these fatalities, and stated that present precautions include all the measures suggested by the experience so far obtained of a new problem, made more difficult by the fact that the manufacturing processes are constantly being modified; he added that further investigations and experiments with new methods and appliances are being made.

### Military Hospitals in India.

The Secretary of State for India was asked by Major Hunt if his attention had been called to the condition of some of the smaller military hospitals in India. Mr. Chamberlain referred the member to previous answers bearing on this subject and to a recent *communiqué* issued to the press; he promised that a report should be called for if any facts were brought forward to warrant it.

Mr. George Lambert was informed of the arrangements being made by the Government of India for the installation of electric cooling-fans and lighting in Barrackpore Hospital and Barracks and elsewhere in India in preparation for the next hot weather.

### Cocaine Prosecutions.

Mr. Brace informed Mr. Raffan that there had been twenty-five prosecutions in the Metropolitan Police District for the violation of the Order in Council prohibiting the sale of cocaine for improper purposes; in none of these cases was the source of supply traced to dental practitioners. Mr. Samuel announced that as a result of a conference with a number of members interested, a small committee would be appointed to make further inquiries as to the use of cocaine by dental practitioners. Pending these inquiries, the temporary permits issued to unregistered dentists will be extended for a short time.

### Sickness Amongst British Troops.

Mr. Forster was asked by Mr. Chancellor to supplement his statement of November 18, 1915, in respect of the incidence of typhoid and paratyphoid fever upon inoculated and uninoculated men serving in France. The Financial Secretary to the War Office stated that up to August 25, 1916, 1,501 cases were finally diagnosed as typhoid fever amongst the British troops in France, 903 amongst inoculated men and 508 amongst non-inoculated men. There were 166 deaths, 47 of which were amongst the inoculated and 119 amongst the uninoculated. To the same date there were 2,118 cases of paratyphoid fever, 1,968 amongst inoculated men and 150 amongst men who had not been inoculated. There were 29 deaths, 22 of which were amongst the inoculated and 7 amongst the uninoculated. Mr. Chancellor further sought similar information as to cases of dysentery, cholera, trench fever, pyrexia, typhoid, paratyphoid, and Gallipoli fever in respect of the Gallipoli Campaign, with the mortality resulting from each. Mr. Forster offered to supply statistics of cases and mortality, but said that no information was available as to how many cases under each category occurred amongst inoculated and uninoculated men respectively.

